# A Chapter Omitted in Most Treatises on General Pathology

**Published:** 1856-11

**Authors:** 


### *A Chapter Omitted in most Treatises on General Pathology.*

[This is the title of a *feuilleton* in a recent number of the *Gazette Médicale*, which dwells so happily on some points bearing upon the reputation and success of the physician, that we have been induced to translate a portion of it. After a playful enumeration of the advantages to be derived from the clinique and the dead room, the paraphernalia of trumpets, tubes, acids, crucibles, lenses, &c., brought to bear on the diagnosis of disease, and portraying the confidence and heartlessness with which the practitioner brings them to the mechanical investigation and treatment of *disease*, quite forgetting that there is a *patient* to be treated also, and indicating the danger that in relying upon the revelations of these, one may be led to neglect questioning a much more important apparatus, the brain—he says:]

But after all, this is not the whole of medicine; science in books, amphitheatres, cliniques, is not true medicine, such as is demanded in the world, in the bosom of society, in the midst of the family. After long and difficult investigation, after scrutinizing facts at the bedside and in the dead room, there is something still higher, still more important and more directly useful; it is the application of all this knowledge so tediously acquired, it is the practical use of the art itself, the direct procedure of the practitioner.

Can any one suppose himself to have become a physician worthy of the name, merely because he has shown an undoubted aptitude for auscultation, percussion, palpation, the analysis of this or that chemical or physical phenomenon; because the use of the lens, or nitric and chlorrohydric acids is child's play to him, and he knows all their reactions? Alas, we have seen in practice manipulators who have passed for masters in all these fine things, who doubted about nothing, who saw the disease *oculorum acie acerrima*, who touched it *non dubitante manu*; but the patient, what became of him, in the hands of these bold explorers?

We must be impressed with the fact that the poor patient, besides flesh and bones, has also a heart, morally speaking—that he hears and strives to comprehend, that he is anxious, that he clings to life by strong attachments, and that our dealing with this living and sensitive being cannot be limited to the mechanical part of the art. It must be recollected that the patient is surrounded by a family which has the greatest interest in knowing what is transpiring, and what is to be expected, and whose solicitude finds in a word, a gesture, a nod of the physician's head, reasons for anxiety, for despair, or for a confidence which the issue does not always warrant.

Here are imperative duties to fulfil, and most important services to render. The man of feeling who is capable of seeing through the surface, who penetrates to the secret fibre, the actual seat of the passions, the spring which maintains the vital movements, the physician who comprehends the extent of his mission, does not limit himself to the material determination of the signs of the malady, and the prescription of the remedies most appropriate for the disease; he goes farther, and sustains the *morale* of the patient, controls him, and exercises over him that salutary influence which the *vir probus medendi peritus* knows how to exert on the mind of the sufferer seeking from him relief and cure.

Listen to Sydenham, who said "*Ægrorum nemo a me alias tractatus est, quam egomet tractari cuperem, si mihi ex iisdem morbis œgrotare contingeret.*" (I treat no patient otherwise than I myself should desire to be treated should I happen to labor under the same disease.) This great man, at the end of his career, was able justly to boast this of himself; we would that every physician should ponder this thought in his heart, and so identify himself with his patient, as to imagine that in treating him he is treating himself—always bearing in mind to have equal respect to the sensibility of the skin and the moral sensibility; to be equally solicitous to assuage physical pain and to administer consolation to the soul—that healing balm which entails blessings upon the hand which bestows it.

Penetrate to the deepest emotions of your patient, put yourself in his place, and see what is to be done. Learn to inspire confidence by the interest you manifest, by patient listening, by assiduous, earnest attendance, by the sympathy he will read in your eye and on your countenance; let him find you at the same time grave and affectionate; avoid with equal care the doleful airs of the fatalist and the frivolous levity and indifference of the sceptic; and, above all, be truthful, that is, say nothing that you do not think, though perhaps not all that you think. God has placed side by side, in the human heart, hope and fear; there is something inhuman, impious, in announcing to the sick what divine prescience has chosen to conceal from them; the physician should throw a favoring veil over the cruel necessity of announcing imminent death, and should take care that the poor incurable shall not have reason to say to him, "I shall apply to another physician; for you neither cure me, nor relieve me, *nor console me.*"—*Boston Med. and Surg. Journal.*

